# Identifying life-history patterns along the fast-slow continuum of mammalian viral carriers

**DOI:** 10.1098/rsos.231512

**Published:** 2024-07-24

**Authors:** Andrea Tonelli, Hernan Caceres-Escobar, Marcus S. C. Blagrove, Maya Wardeh, Moreno Di Marco

**Affiliations:** ^1^ Department of Biology and Biotechnologies ‘Charles Darwin’, Sapienza University of Rome, Rome, Italy; ^2^ Facultad de Medicina Veterinaria y Agronomía, campus Providencia, Universidad de las Américas, Santiago, Chile; ^3^ Institute of Infection, Veterinary and Ecological Sciences, University of Liverpool, Liverpool, UK; ^4^ Department of Computer Science, University of Liverpool, Liverpool, UK

**Keywords:** disease ecology, life-history, viruses, zoonosis

## Abstract

Life-history traits have been identified as major indicators of mammals' susceptibility and exposure to viruses due to evolutionary constraints that link life-history speed with species’ ecology and immunity. Nonetheless, it is unclear where along the fast-slow continuum of mammalian life-history lies the greatest diversity of host species. Consequently, life-history patterns that govern host–virus associations remain largely unknown. Here we analyse the virome of 1350 wild mammals and detect the characteristics that drive species' compatibility with different groups of viruses. We highlight that mammals with larger body size and either very rapid or very slow life histories are more likely to carry different groups of viruses, particularly zoonotic ones. While some common life-history patterns emerge across carriers, eco-evolutionary characteristics of viral groups appear to determine association with certain carrier species. Our findings underline the importance of incorporating both mammals’ life-history information and viruses' ecological diversity into surveillance strategies to identify potential zoonotic carriers in wildlife.

## 1. Introduction

Two-thirds of emerging human diseases are zoonotic [1], and mammals account for approximately 88% of emerging viral zoonoses [2]. Nonetheless, very little is known of the total diversity of the mammalian virome [3], with many unknown zoonotic viruses predicted to circulate in wildlife [4]. Many studies [4–6] tried to uncover the evolutionary dynamics that link mammalian life-history traits to the diversity of viruses that they host. Understanding the patterns that drive viruses’ associations with mammalian species can advance knowledge on host–virus coevolutionary dynamics and direct viral surveillance towards groups of mammals that are more likely to harbour viruses of concern to human and animal health.

A growing body of research in the fields of ecoimmunology and evolutionary biology suggests a fundamental trade-off between immune defenses against pathogens and life-history traits due to the allocation of limited resources within an organism [7–10]. Fast-lived species—with small body sizes, short lifespans, rapid growth and early reproduction—are hypothesized to allocate relatively little energy to immune defences [8]. Probably, these species rely mainly on non-specific innate immune responses against pathogens [9], as these have little metabolic costs for development and maintenance [8]. Fast-lived species might have a higher predisposition to acquire, maintain and transmit viruses due to their weaker defences and tendency to produce numerous offspring that are naïve to infection, which might play an important role in maintaining the infection within the reservoir population [7,11]. By contrast, slow-lived species tend to be large and long-lived, investing more resources into costly defences provided by adaptive immune responses, which are typically slower and pathogen-specific [9]. A large investment in adaptive immunity may be positively selected in slow-lived species, as they are more likely to come into contact with a wide diversity of pathogens multiple times [9].

Several studies have investigated the association between mammalian life-history traits and susceptibility to viruses, reporting mixed evidence as to which of the two ends of the fast-slow continuum is associated with higher propensity to serve as a host [11–18]. Some studies found an association between faster life histories—particularly high offspring production and early sexual maturity—and greater viral competence in mammals [11–13]. But other works provided support of a relationship with slow pace-of-life instead. As an example, zoonotic viral richness in bats has been found to be positively associated with smaller litter size, larger body mass and greater longevity [14–16]. Longer gestation and larger body mass have also been linked to Ebolavirus and Ross River virus seroprevalence across wild mammals [17,18]. In these cases, it has been hypothesized that the immune systems of slow-lived mammals may provide higher tolerance to viral infection, making them suitable hosts for viruses' replication and shedding [9].

Here, we explore the hitherto known mammalian virome to detect patterns of compatibility between mammal species and different groups of viruses, identifying ecological profiles of viral carriers along the fast-slow continuum of mammalian life-history [19]. Our aim is to unveil commonalities and peculiarities across mammalian viral carriers, highlighting common patterns as well as unique features that drive mammals’ susceptibility to individual viral groups. We focus on viral ‘carriers’, instead of ‘hosts’, as the latter implies the species to be competent to the infection and capable of viral shedding, which cannot be assumed from currently available data. We include in the analyses multiple levels of viral complexity by breaking down viral diversity into groups according to three classification schemes: i) structural diversity (based on viral genomic structure and replication), ii) evolutionary diversity (based on viral taxonomy and evolution), iii) ecological diversity (based on a selection of traits that relate to viruses' relationship with their hosts and the environment). We perform separate analyses for all viruses and for the subset of viruses with known zoonotic potential, to highlight any differences in characteristics of general versus zoonotic mammalian carriers.

## 2. Methods

### 2.1. Carrier-virus associations data

We obtained carrier-virus associations from the Global Virome in One Network (VIRION), the most comprehensive database of the global vertebrate virome [20]. We filtered VIRION for unique associations between terrestrial wild mammal species and viruses that allowed for species-level identification of both carriers and viruses (detected by isolation, PCR, or serology). None of the analysed carrier-viral group associations was based on serology alone. Data entries of laboratory and domestic species were removed from our dataset to avoid introducing biased or inaccurate information in our analyses, since experimental infections often represent interactions that do not occur under natural conditions, and domesticated species exhibit life-history traits that have been shaped by artificial selection rather than natural evolutionary dynamics. We identified livestock and domestic species according to FAO's World Watch List for Domestic Animal Diversity [21], while a list of the most common laboratory species used for research purposes worldwide was obtained from the Directive of the European Parliament and of the Council on the protection of animals used for scientific purposes [22] and the Guide for the Care and Use of Laboratory Animals of the United States [23]. The filtering process left us with 9795 unique associations involving 1350 mammal species and 4482 viruses.

**Figure 1.**
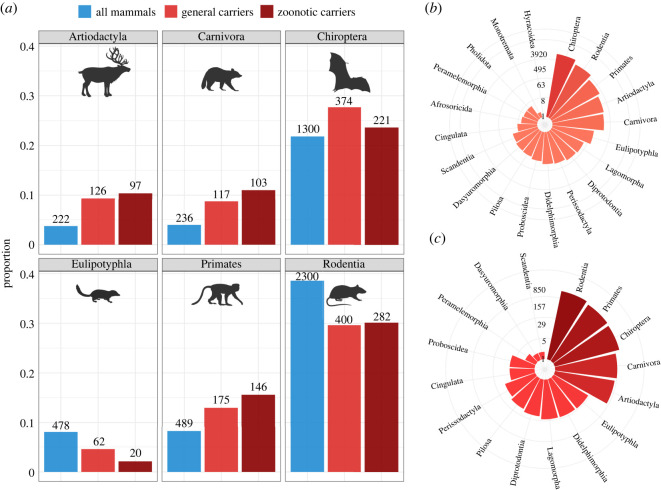
Taxonomic representation of mammalian species in our dataset. (*a*) Comparison between the proportional number of species in the six most represented mammalian orders in the dataset versus their overall relative number. We show the proportion of general (light red) and zoonotic (dark red) carriers represented by a given order, and a comparison with the proportion of mammalian species richness represented by the order (blue). The absolute number of species is shown on top of the corresponding bar. (*b*) Number of unique carrier-virus associations per each mammalian order. (*c*) Number of unique zoonotic carrier-virus associations per each mammalian order. In the circular plots, radial axes are log scaled for representation purposes.

We also identified a subset of 3501 associations between 936 zoonotic carriers and 287 zoonotic viruses (figure 1). Zoonotic carriers were defined as those mammals that share at least one viral species (ratified by the International Committee for the Taxonomy of Viruses, ICTV) with humans according to the VIRION database [20]. Since zoonotic carrier status of mammals was defined using information on viral species (i.e. whether they were zoonotic or not), we only included ICTV ratified species to avoid erroneously including unofficially recognized viral strains and quasi-species. We acknowledge that our definition of zoonotic carriers does not strictly imply competence nor capacity of viral transmission to humans. Integrating experimental data on host competence would help ascertain between susceptibility and competence; however, these data are not currently available for most species and interactions [24].

### 2.2. Carrier characteristics

For each of the 1350 carrier species in the dataset, we collected a set of covariates potentially correlated to carrier status. We obtained life-history characteristics (body mass, longevity, gestation length, litter size, litters per year, interbirth interval and weaning age) from COMBINE, a harmonized repository of published and imputed data for mammal species [25]. To control for environmental confounding factors, we extracted the mean value of ten bioclimatic variables representing temperatures and precipitations within the carrier species' range (electronic supplementary material, table S1). We selected these variables because of their proven ability to predict mammal species distribution at a global scale [26]. All bioclimatic variables were obtained at a spatial resolution of 5 arcminutes (approximately 10 Km at the equator) from the WorldClim database [27]. Species’ range size (km^2^) was obtained from the spatial database of the International Union for Conservation of Nature (IUCN [28]) and included as an additional ecological variable. We also controlled for phylogenetic signals in carrier traits by including ten phylogenetic eigenvectors as indicators of species' phylogenetic position within the mammalian tree of life. The eigenvectors were retrieved from PHYLACINE v. 1.2 [29].

We controlled for correlation among covariates via variance inflation factors (VIF) using the *usdm* package in R [30]. We discarded highly correlated variables (VIF > 9), keeping those that showed relatively higher variable importance during preliminary analyses. Three interrelated bioclimatic variables (i.e. mean annual temperature, temperature of the wettest quarter, and temperature of the driest quarter within the species’ range) were kept as they are usually used to define the essential climatic requirements for mammal species at a global scale [26]. Ultimately, we retained eight ecological variables (range size, mean annual temperature, temperature of the wettest quarter, temperature of the driest quarter, temperature of the warmest quarter, precipitation of the driest quarter, precipitation of the warmest quarter, precipitation of the coldest quarter), and four life-history characteristics (body mass, longevity, gestation length and interbirth interval). A detailed description of all variables is provided in electronic supplementary material, table S1.

### 2.3. Sampling bias

We checked for the existence of bias in the representation of carrier mammalian species included in our dataset. This kind of bias could arise from an unbalanced sampling effort across mammal species, driven by different aspects such as intrinsic biological traits, geographic location, taxonomy and convenience [31]. We found that the statistical distribution of values for the life-history traits we considered did not differ substantially between mammal species in our sample and all existing terrestrial mammal species, meaning that the composition of our sample was not biased in terms of life-history (electronic supplementary material, figure S3). As for taxonomic representation, we found bats to be overrepresented as general carriers, while carnivores, primates and artiodactyls were slightly overrepresented both as general and zoonotic carriers (figure 1). Instead, rodents were proportionally underrepresented as both general and zoonotic carriers (approx. 30%, compared to them representing approx. 39% of all mammalian species).

To account for the different research efforts in identifying viral associations among different species, we counted the number of virus-related publications of each species. The number of publications was retrieved from Web of Science by querying a string made of the scientific binomial of the carrier species AND the terms ‘virus*’ OR ‘viral’. The search was performed in R using the packages *httr* [32] and *jsonlite* [33]. Including this variable in the model allowed us to reduce the effect of differential sampling effort, which might otherwise have influenced our results.

### 2.4. Viral grouping

To have a more detailed view of the complexity of the mammalian virome, we aggregated viral species (42 families) into coherent groups according to three classification schemes: (i) structural, (ii) evolutionary, (iii) ecological (electronic supplementary material, figure S4). Structural groups were derived from the Baltimore classification [34]. The Baltimore classification groups viruses were based on viral genomes' nature and polarity, splitting viruses into seven classes of replication [35]. All genomic information and replication routes needed for viral grouping were retrieved from ViralZone, an online repository of viral bionformatic data [36]. The evolutionary classification reflected the aggregation of viral families into ten phyla, as represented in the ICTV. As for the ecological classification, we split viral families according to traits that have implications for viral ecology, such as evolvability and interaction with the host and the environment. We chose three binary viral traits recognizable at the family level, and then identified seven unique mutually exclusive ecological groups. The selected traits accounted for (i) genomic organization (i.e. linear or circular), (ii) genomic partition (i.e. monopartite or segmented), (iii) pericapsidic envelop (i.e. present or absent). These viral characteristics were chosen as they proved to be relatively important predictors of virus host-range in previous work [37], and were thus able to capture meaningful elements of the interaction between viruses and their carriers. Family-specific viral traits were obtained from ViralZone and Wardeh *et al*. [36]. After grouping viral families together according to the three classification schemes, we identified 7 structural groups, 10 evolutionary groups and 5 ecological groups (totalling 22 groups) (electronic supplementary material, figure S4). We ran separate models for each group, to predict the probability of mammal species to be carriers of viruses in the group. We called these the ‘general models’, as they did not distinguish between zoonotic and non-zoonotic viruses. We then repeated the models after limiting prediction to zoonotic carriers only, thereby defining a set of ‘zoonotic models’. Two viral groups (Preplasmiviricota and Cressdnaviricota) were excluded from zoonotic models due to an insufficient number of carrier species on which to train the models, leaving us with 20 zoonotic models. A complete description of viral grouping is available in the electronic supplementary material (electronic supplementary material, methods).

### 2.5. Definition of non-carrier status

Our analyses relied on confirmed carrier–virus associations, therefore, they lacked any information regarding associations that do not occur in the wild, as no database of unsusceptible associations currently exists [38]. To tackle this issue, we generated pseudo-negatives (i.e. instances of non-carrier status) as substitutes for null interactions for each viral group in both general and zoonotic models. This pseudo-negative design aimed at mimicking virological sampling through an approach based on a combination of taxonomic and spatial cues. Specifically, if the carrier status of a mammal species was unknown, we considered it to be a pseudo-negative whenever the species simultaneously met two criteria: (i) one or more species in the same mammalian family were found susceptible to the viral group (i.e. had a positive carrier status) and (ii) these susceptible species were in the same biogeographical realm as the focal species. This method allowed us to have enough null associations to include in each model, as well as to partially buffer the effect of uneven sampling effort across carrier families. In this respect, we provide estimates of model accuracy for each one of the families included in the analyses in electronic supplementary material, tables S5–S6.

### 2.6. Modelling carrier status

We predicted whether a species had carrier status for different viral groups as a function of the above-described variables. We used random forest models for analysis, a powerful machine learning technique capable of modelling complex interactions among predictors [39]. We ran 22 classification models to assess general carrier status and 20 classification models to assess zoonotic carrier status, where each model targets a different viral group. As species tended to be unevenly distributed among the two responses' classes (carrier versus non-carrier), we assigned different weights to each observation to avoid bias in predictions towards the overrepresented class. Hence, in every model, we assigned each observation a weight equal to the number of observations of the opposite class.

We used a 10-fold cross-validation and a tuning grid to train each model and find the optimal combination of two hyperparameters: the number of randomly selected variables available for splitting at each tree's node (*mtry*) and the minimal number of observations required for a node to be split further (*n_min*). The 10-fold cross-validation divided the training set (75% of total data) into 10 random samples and iteratively held one out to use it for testing. We repeated the iteration process using different combinations of hyperparameters, then kept the combination that yielded the best performance (quantified by the true skill statistic metric, *TSS*) to obtain the final model. TSS is calculated as follows:

2.1 TSS=Specificity+Sensitivity−1,

where sensitivity represents the proportion of true positives, i.e. carrier species that are correctly classified as such, and specificity is the proportion of true negatives, i.e. non-carrier species that are correctly classified as such. TSS ranges between −1 and +1. A TSS equal to +1 denotes perfect classification performance while a TSS equal to −1 denotes perfect misclassification [40].

The predictive performance of each final model was then evaluated in two distinct ways. First, we calculated the models' *TSS* by predicting the carrier status of species in the test set (25% of total data) and comparing the predictions with the observed carrier status. Then, we performed a taxonomic block validation at the family level. This validation method works by iteratively holding out a target group of observations (in this case, each mammalian family) from the training set, to use it for model testing. Families with fewer than ten observations per class were not included in the validation pipeline. In addition to making training and testing sets independent, the block validation allowed us to assess the generalizability of our results across different carrier taxa. Based on the cross-validation performance, we excluded from analysis models with a low *TSS* value (<0.30).

From the models’ outputs, we extracted the estimate of the importance of each variable in predicting carrier status for the different viral groups. We quantified variable importance as the mean decrease accuracy of the model, i.e. the decrement of the accuracy of model predictions caused by the variable's permutation, averaged across all trees. A larger value indicated a relatively more important predictor, as its exclusion led to a bigger loss of model accuracy. Lastly, the variables' importance scores (*x*) obtained from the models were rescaled within classification schemes to allow comparison between viral groups, according to:

2.2 $$x_{\mathrm{norm}}=\;\frac{x-x_{\min}}{x_{\max}-x_{\min}}.$$

We used partial dependence plots to assess the relationship between the predicted probability of a species being a general/zoonotic carrier and selected species’ characteristics, according to each model. To obtain the partial dependence on a selected variable, the outcome of the model was marginalized over the distribution of the remaining variables, so that the resulting function depended only on the targeted predictor [41]. Then, the functions were plotted through locally estimated scatterplot smoothing (*LOESS*) curves, to display the average trend of carrier status probability given different values of the explanatory trait. We used the R package *ranger* [42] and the R suite *tidymodels* to run and validate the models [43].

## 3. Results

### 3.1. Model accuracy

We used a set of life-history, ecological, and bioclimatic covariates to assess carrier status of 1350 wild mammalian species, totalling 9795 general carrier–virus associations and 3501 zoonotic carrier-virus associations (figure 1, electronic supplementary material, figures S1–S2). We ran 22 general models (including all viruses) and 20 zoonotic models (including just zoonotic viruses) to identify how mammalian species traits affect the probability of being carrier species of different structural, evolutionary and ecological viral groups. Eight models (five general and three zoonotic) were excluded from further interpretation due to low performance (*TSS* < 0.30) during cross-validation.

General and zoonotic models showed moderate accuracy during cross-validation, with a *TSS* of 0.42 (range: 0.31–0.53) and 0.46 (range: 0.32–0.74) respectively. As expected, when assessed with a taxonomic block validation, general and zoonotic models showed a lower average accuracy of 0.22 (mean range: 0.04–0.39) and 0.19 (mean range: 0.00–0.40), respectively. Some carrier families exhibited noticeably low performance during taxonomic block-validation, compared to cross-validation, suggesting that certain family-specific patterns have not been effectively captured by our models. To test whether filtering models based on the taxonomic block validation would have affected the patterns found in this work, we ran a sensitivity test on a subset of our dataset (Baltimore class I). Importantly, our test demonstrates that the shapes of relationships between carrier status and key life-history traits do not change substantially when using the more stringent inclusion criteria (taxonomic block-validation threshold: TSS > 0.3) (electronic supplementary material, figure S5). Model performance metrics extracted from both cross-validation and block-validation processes are available in electronic supplementary material, tables S3–S6.

### 3.2. Characteristics of general carriers

**Figure 2.**
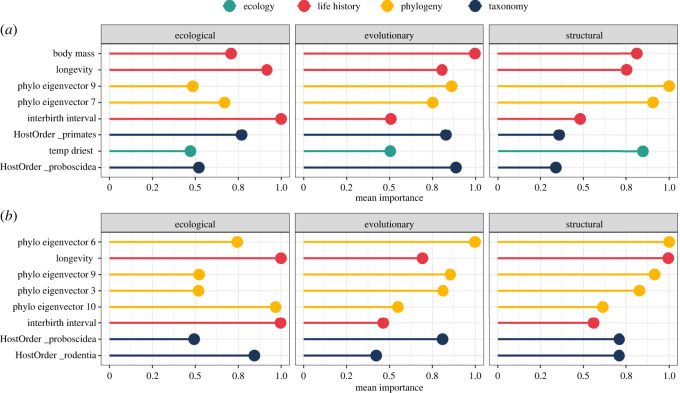
Mean variable importance of the mammalian traits used to assess (*a*) general and (*b*) zoonotic carrier status within ecological, evolutionary, and structural viral classification schemes. The importance of each variable is quantified as the mean decrease accuracy of models' predictions caused by variable random permutation. Mammalian traits are further classified visually by ecology, life history, phylogeny and taxonomy. Variable importance scores outputted by each model, for each viral group, were rescaled between 0 and 1. Here we only show the most important variables across the various schemes (see electronic supplementary material, figures S5–S6 for a full representation of all predictors). For representation purposes, we excluded research effort (quantified as the number of virus-related citations) which we used to account for sampling bias in our dataset.

After accounting for research effort, body mass was the most important predictor of general carrier status across most viral groups in the three classification schemes. The next most important predictors were indicators of species' phylogenetic position (i.e. phylogenetic eigenvectors), followed by species’ longevity and interbirth interval (figure 2).

**Figure 3.**
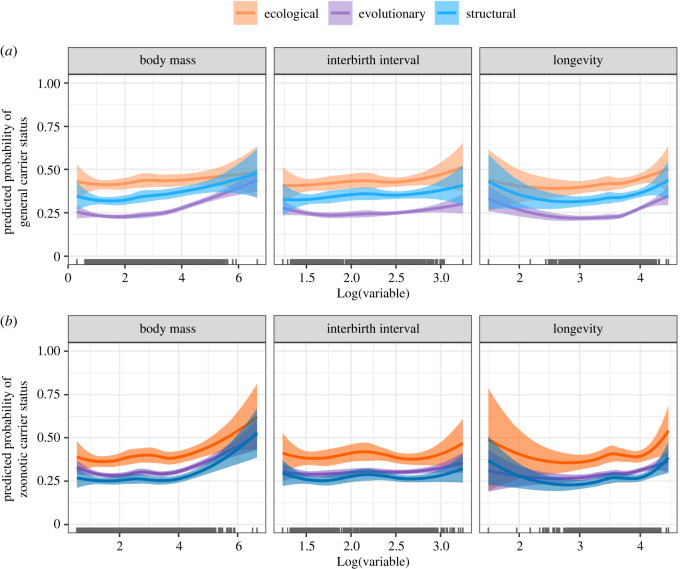
Mean variable importance of the mammalian traits used to assess (*a*) general and (*b*) zoonotic carrier status within ecological, evolutionary and structural viral classification schemes. The importance of each variable is quantified as the mean decrease accuracy of models’ predictions caused by variable random permutation. Mammalian traits are further classified visually by ecology, life history, phylogeny and taxonomy. Variable importance scores outputted by each model, for each viral group, were rescaled between 0 and 1. Here we only show the most important variables across the various schemes (see electronic supplementary material, figures S5–S6 for a full representation of all predictors). For representation purposes, we excluded research effort (quantified as the number of virus-related citations) which we used to account for sampling bias in our dataset.

The relationships between mammals' life-history traits and carrier status were largely consistent across viral groups, with few exceptions. The relationship between body mass and the probability of being a carrier was typically positive (figure 3), with a rapid increase in probability for species larger than 0.3–3 kg depending on the viral group considered (electronic supplementary material, figure S8). The relationship appeared stronger with viruses in the 4th ecological group (non-enveloped viruses with a linear segmented genome), the 3rd Baltimore class (dsRNA viruses) and the phyla Duplornaviricota, Peploviricota and Nucleocytoviricota. Species’ longevity showed an idiosyncratic effect on carrier status, with species on both extremes of mammals' lifespan range more likely to be predicted as carriers of several viral groups (figure 3). This average trend was mostly driven by the negative response of Cressdnaviricota carriers as well as the bimodal relationship between longevity and carrier status for the 3rd ecological group (enveloped viruses with a linear non-segmented genome), the 2^nd^ Baltimore class (ssDNA viruses), and the phylum Cossaviricota (electronic supplementary material, figure S9). In the remaining groups, long-lived species were associated with a higher probability of being carriers. As for interbirth interval, its effect on carrier status probability was generally positive (figure 3), although the 6^th^ (enveloped viruses with a linear segmented genome) and 1^st^ (non-enveloped viruses with a linear non-segmented genome) ecological groups, and the phylum Cressdnaviricota showed an opposite (negative) trend (electronic supplementary material, figure S10).

### 3.3. Characteristics of zoonotic viral carriers

Once research effort was accounted for, zoonotic carrier status for most viral groups was best predicted by two of the life history traits already identified as drivers of general carrier status—longevity and interbirth interval—together with species’ phylogenetic position (figure 2). In most viral groups, the relationship between life-history traits and zoonotic carrier status showed the same qualitative trends as in general carriers, but with a comparatively stronger quantitative effect. This pattern was evident in the average relationship between body mass and zoonotic carrier status, which showed more accentuated upward trends compared to that of general carriers (figure 3). Similar to general carrier models, longevity showed an idiosyncratic relationship with zoonotic carrier status probability. The relationship between interbirth interval and zoonotic carrier status was also similar to that of general carriers, with more likely reservoirs falling on both extreme ends of the variable's range. This pattern was consistent across most viral groups, with the exceptions of the 1st (non-enveloped viruses with a linear non-segmented genome) and the 6th (enveloped viruses with a linear segmented genome) ecological groups where the effect was negative, and the 3^rd^ ecological group (non-enveloped viruses with a circular non-segmented genome) where the effect was strongly positive (electronic supplementary material, figure S10).

## 4. Discussion

Despite high scientific interest in the ecological and evolutionary drivers of mammal–virus associations, contrasting evidence exists on the characteristics that make wild mammals more subject to acquiring and transmitting viruses. Once research effort and phylogeny are accounted for, our results show that the probability of mammals' carrier status for different viral groups is best predicted by species’ body mass, longevity, and interbirth interval, although variable importance in some groups showed a substantial difference from the others. Such differences might be explained by group-specific patterns of associations with mammalian carriers as well as unaccounted for sampling biases in carrier-viral group associations. Our models predicted large-sized mammals on both extreme ends of the fast-slow continuum of life-history as being more likely to carry viruses. These results suggest that although mammals on opposite ends of the fast-slow continuum have likely evolved diverging immunological strategies, viruses are able to exploit both strategies for replication and spread. This means that the peculiarities of each viral group may drive patterns of ‘carrier preference' reflecting viruses' replication speed, adaptability and possibly evolutionary history.

Mammalian species with larger body sizes had higher carrier probability for most general and zoonotic groups. Evidence from comparative studies in the field of eco-immunology suggests that elements of immunity [44], and pathogenesis [45] scale with body size. It has been hypothesized that larger species evolved greater tolerance to infection to minimize the costs of infection in favour of survival, which may, in turn, make them more capable of being viral carriers. Still, the mechanisms driving the relationship between body mass and immunity phenotypes remain uncertain and their effect on virus suitability and tolerance are open to question [46].

Additionally, large-bodied species have higher dispersal abilities and need large home ranges in order to meet their high metabolic requirements [47]. The ability to move farther may play a role in increasing exposure to viral infections, as these species are more likely to come into contact with disease hosts (e.g. primary, secondary or reservoirs) and vectors. Past works have found mammals' body mass to be related to susceptibility to tick-borne encephalitis virus, yellow fever virus, and Zika virus [48], suggesting that large-bodied species might significantly contribute to vector-borne viruses' transmission. Moreover, home range was a key predictor of wildlife hosts of Rift Valley fever virus [49], in agreement with the hypothesis that larger home ranges may enhance exposure to viruses by increasing inter- and intraspecific contacts. Large-sized mammals also consume large amounts of food to meet high energetic requirements [50], which makes them more likely to ingest viruses that rely on indirect routes of transmission via fomites (such as contaminated food sources).

In addition to the aforementioned mechanisms of susceptibility and exposure to viral infection, the larger body mass of zoonotic carriers may be explained by dynamics that underlie zoonotic viral spillover from wildlife. Humans interact more frequently with large wild mammals through several forms of direct exploitation, such as hunting and wildlife trade [51], and hunters typically have a preference towards large-bodied target species [52–54]. Practices such as wildlife hunting and trade may pose a public health risk because of the often-lacking sanitary controls and inadequate animal manipulation [55], which may facilitate viral spillover to people at different stages of the supply chain. Large mammals may also contribute to the emergence of zoonotic viral diseases as amplification hosts (i.e. organisms in which the infectious agent can replicate rapidly and reach high concentrations) [56]. It is possible that large mammals can develop higher viral loads and release greater quantities of virus, increasing the probability of transmission in case of encounter with humans or other species [17]. Traditionally, livestock animals are considered to be amplification hosts for zoonotic viruses [57,58], but evidence of wild amplifiers has been found for Old World monkeys as chikungunya virus hosts in Senegal [59], great apes and forest antelopes as Zaire Ebola virus hosts in West Africa [60], and white-tailed deer as Cache Valley virus hosts in the United States [61].

In both general and zoonotic models, mammals placed on the slower end of the life-history continuum (longer longevity and interbirth intervals) were more likely to have carrier status for relatively slow-evolving viral groups associated with chronic or persistent infection, such as RNA retroviruses (phylum Artverviricota) and dsDNA viruses (phyla Peploviricota, Baltimore classes VII and I). To ensure transmission, most dsDNA viruses and retroviruses are required to remain in the host for extended periods of time, resulting in persistent or chronic infections with low or delayed disease severity and virulence [62]. Because of the extended infectious period, viruses that cause chronic or persistent infection (e.g. Herpesvirus, Lentivirus) are expected to have a greater viral fitness (i.e. the capacity of a virus to produce infectious progeny) in long-lived hosts [15]. Our results sustain this hypothesis, suggesting that viruses which stay in the host for longer got a selective advantage by co-evolving with—and adapting to—slow-lived species which may carry the infection for prolonged periods of time and increase the probability of transmission.

For general and zoonotic carriers of viruses with linear genomic segments and an envelope (i.e. those in our 6th ecological group, such as Arenaviridae, Hantaviridae and Peribunyaviridae), carrier status probability was highest for species with faster life-history as represented by short longevity and interbirth interval. These RNA viruses, which include Lassa fever virus and Sin Nombre virus, are considered relatively fast-evolving due to rapid nucleotide substitution rate and ability to reassort homologous genome segments [62,63]. Our findings suggest that such evolutionary mechanisms may be further favoured in fast reproducing carriers, where population density and generation overlap may increase viral fitness due to the large availability of naïve individuals. Still, we cannot fully exclude that these results were driven by taxa representation in the training set, as bats, moles, shrews and especially rodents—that are considered the main natural hosts of these viruses—display shorter lifespans and faster life-histories. To further explore the adaptive links between viral evolvability and host traits it will be necessary to address critical data gaps on host–virus interactions which currently limit our ability to make inference.

It is important to consider that our study has limitations, associated with the available data on mammal-virus associations. We defined mammal-virus associations as the detection of a virus in a given mammalian species via PCR, isolation or serology. These detection methods do not necessarily imply that the species is an actual viral reservoir, rather, they inform us that the species is susceptible to infection. Thus, it is likely that we included different levels of susceptibility within the term ‘carrier’, such as: reservoir, natural host, and incidental host. Additionally, we recognize that sampling bias is present in the data, as the observed patterns of viral distribution across host taxa are not an accurate representation of the—largely unknown—mammalian virome [64]. Such limitations affect any study investigating general host-pathogen associations from a limited number of observations, as even well-known taxa of zoonotic carriers (such as rodents) remain understudied. For example, due to the underrepresentation of rodents as carrier species, our models might have not detected patterns that involve combinations of life-history traits typical of rodents, such as small body mass, short lifespan, and high offspring production. By working above the viral species level using dichotomous outcomes (carrier status), and accounting for species' virus-related citations (i.e. reporting effort), we mitigated the impact of such bias.

In this work, we identified common patterns along the fast-slow continuum of mammalian viral carriers, highlighting that mammals' associations with viruses are generalizable only to a certain extent. The eco-evolutionary peculiarities of each viral group drive patterns of compatibility between mammals and viruses, as we partially captured by separately assessing different eco-evolutionary groups of viruses. By including multiple sides of viral diversity, we were able to pick up on trends that went undetected in previous studies where pathogen richness was analysed as a whole [4,65]. We found that larger mammals are carriers for several viral groups, but both fast- and slow-living species are exploited by multiple viral groups with different ecological, evolutionary and structural features. General and zoonotic carriers' profiles did not differ substantially in terms of life-histories, providing new evidence in support of the hypothesis that there are no intrinsic traits that disproportionately affect mammals' susceptibility to zoonotic viruses [66]. We argue that underestimating viruses' functional diversity may lead to neglecting potentially important sources of zoonoses, with the risk of impairing hazard assessments and outbreak preparedness. Using the insights provided by life-history theory may enhance surveillance strategies in areas where species with a higher proneness for carrying viruses are more abundant.

## Data Availability

No new data were created in this study. Code to reproduce the study: https://doi.org/10.13133/UNIROMA1/VXMHCX. The data are provided in electronic supplementary material [67].
